# Assessing the Cloud-RAN in the Linux Kernel: Sharing Computing and Network Resources

**DOI:** 10.3390/s24072365

**Published:** 2024-04-08

**Authors:** Andres F. Ocampo, Mah-Rukh Fida, Ahmed Elmokashfi, Haakon Bryhni

**Affiliations:** 1 SimulaMet—Simula Metropolitan Center for Digital Engineering, 0167 Oslo, Norway; haakonbryhni@simula.no; 2 Faculty of Technology, Art and Design, OsloMet—Oslo Metropolitan University, 0176 Oslo, Norway; 3 School of Computing and Engineering, University of Gloucestershir, Cheltenham GL50 2RH, UK; mrukh@glos.ac.uk; 4 Amazon Web Services (AWS), Seattle, WA 98109, USA

**Keywords:** Cloud radio access network (Cloud-RAN), functional splitting, vRAN, 5G cellular networks, RT Linux

## Abstract

Cloud-based Radio Access Network (Cloud-RAN) leverages virtualization to enable the coexistence of multiple virtual Base Band Units (vBBUs) with collocated workloads on a single edge computer, aiming for economic and operational efficiency. However, this coexistence can cause performance degradation in vBBUs due to resource contention. In this paper, we conduct an empirical analysis of vBBU performance on a Linux RT-Kernel, highlighting the impact of resource sharing with user-space tasks and Kernel threads. Furthermore, we evaluate CPU management strategies such as CPU affinity and CPU isolation as potential solutions to these performance challenges. Our results highlight that the implementation of CPU affinity can significantly reduce throughput variability by up to 40%, decrease vBBU’s NACK ratios, and reduce vBBU scheduling latency within the Linux RT-Kernel. Collectively, these findings underscore the potential of CPU management strategies to enhance vBBU performance in Cloud-RAN environments, enabling more efficient and stable network operations. The paper concludes with a discussion on the efficient realization of Cloud-RAN, elucidating the benefits of implementing proposed CPU affinity allocations. The demonstrated enhancements, including reduced scheduling latency and improved end-to-end throughput, affirm the practicality and efficacy of the proposed strategies for optimizing Cloud-RAN deployments.

## 1. Introduction

Considered a key enabler for the upcoming generations of mobile systems, the Cloud-RAN architecture presents a compelling evolution of the RAN design [1]. By leveraging both software-defined wireless networking and virtualization technology, Cloud-RAN enables the deployment of multiple vBBUs on centralized edge servers, providing operational and maintenance benefits for mobile network operators.

To manage latency-critical vBBU functions, the host edge server employs a real-time (RT) Operating System (OS) that offers guarantees to meet the specific latency requirements of these tasks. However, substantial costs related to the development, maintenance, and licensing of an RTOS have led to a shift towards adopting Linux—originally designed as a general-purpose OS-for managing RT systems [2,3]. The Linux Kernel incorporates several RT mechanisms such as RTLinux [4], the low-latency patch [5], and the PREEMPT_RT patch [6], enabling it to support diverse RT systems, including vBBUs [7]. Throughout this paper, we refer to a Linux system equipped with these RT functionalities as the Linux RT-Kernel.

Within the Linux RT-Kernel, the vBBU executes a combination of RT processes for latency-sensitive tasks [8], including physical layer functions, alongside non-RT processes managing latency-tolerant tasks such as control layer functionalities [9]. Despite the RT-Kernel ability to prioritize RT processes, the performance of latency-critical vBBU functions might be potentially impacted by computation-intensive workloads running in the user-space, as well as from non-preemptible Kernel threads, especially when they are scheduled on the same CPUs. Furthermore, in the advent future generation of mobile systems like 6G and the proliferation of IoT devices, the mobile network architecture must support an ever-increasing number of latency-critical applications. This requires an in-depth understanding of resource sharing implications to maintain stringent performance requirements.

While the existing literature suggests that vBBUs can be effectively run on general-purpose servers [10], the detailed operational implications of such shared-resource scenarios remain not fully understood [11]. Our study seeks to bridge this gap by investigating how compute-intensive workloads and Kernel thread activity influence vBBU performance. Moreover, we assess the effectiveness of CPU management methodologies such as CPU affinity [12] and CPU isolation [13], which are established techniques used to enhance the performance and stability of RT systems in general-purpose computing environments [14,15,16,17].

This study offers a focused empirical analysis of deploying vBBUs on general-purpose edge servers managed by the Linux RT-Kernel, with a specific emphasis on the performance implications of resource sharing in Cloud-RAN deployment. Although our analysis is concentrated on this niche, it serves as a foundation for further inquiry into various facets of vBBU performance degradation in different operational contexts. Our contributions are twofold. Firstly, we provide an empirical examination of resource sharing for latency-critical systems like vBBUs on general-purpose edge servers, particularly relevant in emerging generations of mobile systems such as 5G and potential 6G architectures [18,19]. Secondly, we investigate the applicability of CPU affinity and isolation strategies in mitigating performance degradation in Cloud-RAN environments managed by the Linux Kernel. This work advances the understanding of vBBU operations in shared environments, crucial for optimizing Cloud-RAN implementations and ensuring consistent vBBU performance across diverse computational settings.

The rest of the paper is organized as follows. Section 2 presents the background and related work. Section 3 discusses the system model used to deploy vBBU on edge servers. In Section 4, we assess the performance of latency-critical vBBU processes in the Linux RT-Kernel when sharing computing and network resources. This section also discusses strategies for mitigating processing interference from collocated workloads. Section 5 evaluates the performance in the Cloud-RAN architecture, focusing on shared computing and network resources among vBBUs. Lastly, Section 6 concludes the paper.

## 2. Background and Related Work

This section provides an overview of the Cloud-RAN architecture and highlights the execution of vBBUs on MEC servers managed by the Linux RT-Kernel.

### 2.1. The Cloud-RAN Architecture

**Figure 1:**
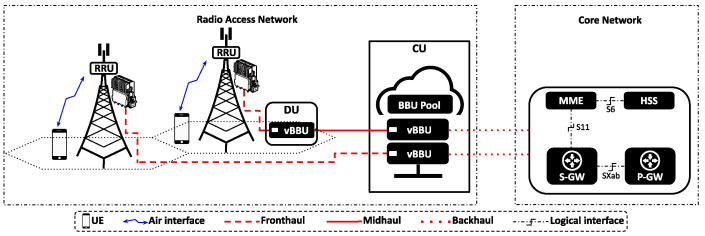
Cloud Radio Access Network architecture.

The RAN comprises the User Equipment (UE), the air interface, antennas, the Remote Radio Units (RRUs), the BBU, and a network link connecting the RRU and the BBU, known as Fronthaul. The RAN is connected to the Core Network (CN) via a transport network called Backhaul. As depicted in Figure 1, within Cloud-RAN, the BBU is implemented as a software-defined wireless networking application (vBBU). By leveraging virtualization technologies, multiple vBBUs can be deployed on a centralized MEC server, sharing both processing and network resources [1].

The Cloud-RAN architecture presents a new paradigm for RAN design by enabling the sharing of computing and networking resources. Nevertheless, the centralization of vBBUs brings about stringent latency constraints and capacity demands for both the Fronthaul network and the MEC server that houses the vBBUs. In response to these challenges, the 3GPP has proposed a functional split of the vBBU protocol stack [20].

Processing a portion of the vBBU functions locally near the antennas helps alleviate bandwidth and latency demands on the Fronthaul [21]. This approach is supported by the IEEE 1914 working group that has defined two logical split point placements [22]: the Distributed Unit (DU), near the cell tower, and the Centralized Unit (CU), based on the Mobile Network Operator’s edge server. The introduction of these split points reclassified the mobile transport network segments and their respective latency and capacity requirements [23]. The Fronthaul is the segment between the RRU and the DU, the Midhaul connects the DU and the CU with data rate requirements that vary based on the selected functional split, and the Backhaul connects the Cloud-RAN with the CN. Collectively, these transport segments constitute the mobile Crosshaul (Xhaul).

**Figure 2:**
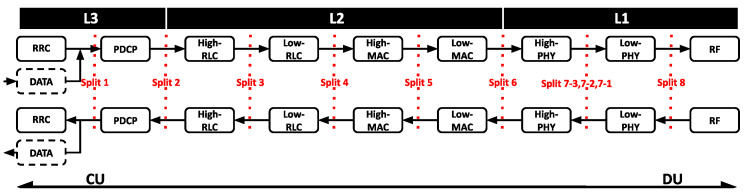
3GPP functional split of the LTE–BBU functionality [20].

As illustrated in Figure 2, the dotted red line signifies the split option as defined by the 3GPP [20]. In this model, L1 corresponds to physical layer functions (such as low PHY and high PHY), L2 identifies link layer functions (such as MAC and RLC), and L3 is associated with network layer functions (such as PDCP and RRC). Functions to the left of a given option are instantiated at the CU, while functions to the right are allocated to the DU. The more functions designated to the DU, the less stringent the latency and capacity demands.

### 2.2. Hosting vBBU on MEC Servers

While L1 and L2 functions perform signal processing with RT requirements, L3 functions do not have timing demands. Therefore, a vBBU operates as a multi-process application within an OS, composed of a mix of RT and non-RT processes [24]. To meet the timing requirements of the RT processes on a MEC system, the host OS must provide RT guarantees. This includes preemption and a scheduling policy that prioritizes meeting the timing constraints of individual processes over maximizing the average number of scheduled processes.

#### 2.2.1. Linux as the Host OS for Running vBBUs

Over recent years, several mechanisms have emerged to provide RT support within the Linux Kernel. Key examples comprise of RTLinux [4], the low-latency patch [5], and the PREEMPT_RT [6] patch. This development has positioned Linux to be used in RT systems [25], particularly in the domain of RT signal processing for vBBU functions [7]. For instance, Linux RTAI (Real-Time Application Interface) [26] has found applications in mobile system testbeds [7,27,28,29,30]. Studies in [29,30] demonstrate the performance of vBBUs using the PREEMPT_RT patch in the context of Cloud-RAN. The Low-Latency Kernel patch [5], integrated into the mainline code of the Ubuntu distribution, has gained widespread adoption, particularly among researchers using the OAI code [31,32,33]. This popularity stems from the optimization of OAI’s code for seamless compatibility with the Low-Latency Kernel.

#### 2.2.2. Virtualization Environments with RT Support for vBBU Execution

Virtualization technology, such as hypervisors and containers, enables the concurrent execution of multiple vBBUs on the same edge server as isolated processes. In hypervisor-based virtualization, the hypervisor dictates the RT scheduling mechanism that allocates CPU time to virtual machines (VMs). Simultaneously, the guest OS must implement an RT-Kernel capable of preempting non-RT tasks in favor of RT ones [34]. In contrast, for containerized virtualization, the edge server adopts an RTOS with preemption and an RT scheduling mechanism. This scenario requires containers to link their binaries and libraries to the host’s RTOS [35].

Containers, as demonstrated in studies like [36], offer superior RT performance compared to VMs, primarily due to reduced overhead from both the hypervisor and the guest OS. For example, investigations in [37,38] explored various virtualization environments within the context of Cloud-RAN, encompassing hypervisors, Docker containers, and Linux containers (LXC). These studies compared them with a bare-metal deployment and found that containers exhibit shorter processing times than hypervisor VMs, with LXC demonstrating processing times similar to those of a bare-metal deployment. Recently in the field of virtualization technology, specifically in containerized virtualization, RT containers [39] have emerged to support RT applications. However, research on the performance outcomes of RT applications running on containers, specifically when sharing resources with collocated workloads, is still required. Likewise, while prior research has validated the feasibility of operating vBBUs on edge servers [32,40], it is critical to understand their performance in scenarios where they concurrently utilize computing resources with collocated workloads [41].

### 2.3. Resource Sharing in MEC Servers

When running the vBBU on a MEC system managed by the Linux RT-Kernel, vBBU’s processes may share computing resources with either collocated user-space processes or Kernel threads. This situation could potentially result in collocated workloads causing processing interference for RT processes. Such interference may stem from the sharing of physical resources [42,43] or Kernel space processing [44,45], both of which could have a detrimental impact on the performance of RT processes [46].

Sharing physical resources, such as CPU, I/O, and memory, among applications with various execution time requirements (e.g., mixed time-critical services—MCS), on general-purpose servers has been extensively studied in the academic literature [47]. However, only a few pieces of research have explored the processing interference resulting from Kernel/user space processing. For instance, a study by Reghenzani et al. in [45] analyzed the processing interference caused by different Kernel subsystems under a variety of workloads related to MCS.

In the context of MCS in embedded systems, a common strategy to mitigate processing interference with collocated applications entails running delay-sensitive services as RT processes on a set of isolated CPUs [43,48]. However, this approach may not align seamlessly with the shared computing resources and multi-tenancy characteristics of Mobile Edge Computing [49]. According to a recent study [50], CPU utilization by a vBBU during peak times does not go beyond 60%, suggesting the possibility of resource sharing. As such, the authors suggested a CPU allocation mechanism for vBBUs that share CPU resources with collocated workloads, intending to minimize CPU underutilization.

Despite these findings, further research is needed to fully understand the performance implications of resource sharing in edge servers, particularly when running RT applications concurrently with other workloads. While existing studies have examined vBBU performance across different OS and virtualization environments, our research provides a comprehensive investigation by delving into the effects of resource sharing on vBBU performance within the Linux RT-Kernel. This methodology enables us to evaluate not only the overall mobile system performance but also the individual procedures within the vBBU.

## 3. System Model: Instantiating the vBBU within the Linux Kernel

This section delves into the instantiation of vBBU within the Linux RT-Kernel, discussing both CPU affinity and CPU isolation as strategies to mitigate potential processing interference that may arise from resource sharing with collocated workloads.

### 3.1. Mobile Network Scenario

**Figure 3:**
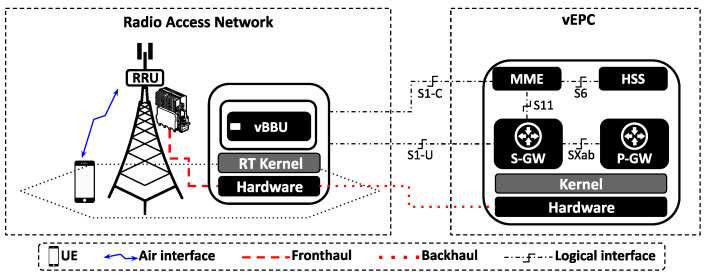
Mobile network scenario: monolithic vBBU running on a bare-metal GPP managed by Linux RT-Kernel. The vEPC is deployed on bare metal GPP managed by Linux generic Kernel.

Figure 3 depicts the mobile network scenario considered in this paper, which includes a single user equipment (UE), a monolithic vBBU, and the core network (CN). The vBBU is hosted on a MEC server with eight CPUs, managed by the Linux RT-Kernel. The CN, on the other hand, consists of a software implementation of the Evolved Packet Core (vEPC), instantiated on a general purpose server. For detailed software and hardware specifications used in the experimental setup corresponding to the mobile network scenario, please refer to Appendix A.

### 3.2. Instantiating the vBBU within the Linux RT-Kernel

When instantiated in the Linux RT-Kernel, a vBBU operates as a multi-threaded user-space process, incorporating a mix of both RT and non-RT threads. The method of instantiation for these threads can vary subject to the specific implementation, such as OpenAirInterface (OAI) [51] and SrsLTE [52]. This paper centers around the LTE–eNB implementation by OAI, utilized as the vBBU. This LTE–eNB implementation, when operating under the Linux RT-Kernel, initiates a selection of RT threads and non-RT threads as depicted in Table 1.

The subset of RT threads is responsible for embedding and processing the L1 and L2 functions of the vBBU within the Linux RT-Kernel. These threads are designated as RT user-space threads due to the strict timing requirements associated with their operations. Such strict timing constraints are critical, for instance, in adhering to the Hybrid Automatic Repeat reQuest deadline [32]. In particular, the ru-thread ($\tau_{1}$) handles low-level L1 functions, primarily dealing with tasks such as reading from and processing signals for the RRU receiver and processing and writing signals for the transmitter. Likewise, the fep_processing thread ($\tau_{3}$) handles L1 functions through the Front End Process. This involves defining procedures for the Uplink (UL) and processing precoding and Single Carrier Frequency Division Multiple Access signals. For the Downlink (DL), the feptx_thread ($\tau_{4}$) serves as the FEP for TX and manages DL procedures, pre-coding, and Orthogonal Frequency Division Multiplexing signal processing [40]. Meanwhile, the lte-softmodem thread ($\tau_{2}$) takes on the processing of the remaining L1 functions along with L2 functions.

Conversely, the non-RT thread subset includes L3 processing functions and control procedures such as RRC, STCP, and S1AP. Also categorized as non-RT threads are functions tasked with processing user data plane traffic through the GTP tunnel, an example of which is TASK_GTPV1_U ($\tau_{5}$). This uses the transport protocol UDP, as seen in tasks like TASK_UDP ($\tau_{6}$).

### 3.3. Sharing Computing Resources with User-Space Workloads and Kernel Threads

As the vBBU may share computing resources (e.g., CPU time, I/O, memory) with user-space processes and Kernel threads, understanding the impact of such resource sharing on vBBU performance is crucial. To this end, three vBBU execution scenarios are considered.

The first scenario, denoted *Idle*, involves running the vBBU with no collocated user-space processes or Kernel threads. The second scenario, denoted *User*, entails running the vBBU concurrently with other user-space processes on the same set of CPUs. This setting aims to replicate a situation where the vBBU shares computing resources with non-RT user-space processes. To emulate this, workloads are generated on various subsystems of the edge server. For instance, user-space processes are executed on each CPU, simulating intensive I/O operations, virtual memory stress, disk read/write operations, and message sending/receiving using POSIX. This approach offers a thorough assessment of how the vBBU performs amid the strain of user-space collocated workloads, providing valuable insights into potential challenges and performance implications in real-world scenarios. In the third scenario, named Kernel, the vBBU operates alongside Kernel threads on the same set of CPUs. Kernel threads, serving as entities utilized by the Kernel for CPU-time allocation, are non-preemptive [53]. This characteristic introduces the potential for an impact on vBBU performance if Kernel threads are scheduled on the same CPU [25]. To delve into the influence of Kernel thread processing on vBBU performance, this paper narrows its focus to Hard-IRQs generated when the Linux RT-Kernel receives incoming network packets [54].

Upon receiving a packet, the network interface card (NIC) driver initiates a Hard-IRQ, signaling the availability of an incoming packet for processing. In situations where there is a high flow of incoming packets to the NIC, a corresponding surge in Hard-IRQs occurs. Handling a large number of Hard-IRQs can monopolize CPU time, potentially resulting in starvation for other processes [55]. To address this, the Linux RT-Kernel uses an adaptive mitigation mechanism known as the New API [56]. NAPI suppresses the generation of Hard-IRQs when the number of incoming packets on the NIC exceeds a pre-defined threshold, known as the NAPI weight. As an alternative, NAPI generates software IRQs [57]. These Soft-IRQs systematically poll and process packets in batches, with the batch size matching the NAPI weight. Furthermore, the NAPI’s Soft-IRQ handler runs within the context of the NIC’s Hard-IRQ handler (i.e., Kernel thread) that triggers the Soft-IRQ [54]. When the count of received packets drops below the NAPI weight threshold, the NIC’s driver disables the NAPI mechanism and resumes the generation of Hard-IRQs.

### 3.4. Enhancing RT Performance for vBBU Processes with CPU Affinity

The RT-Kernel’s Scheduler is designed to maximize the allocation of CPU time across numerous tasks, accomplished by frequently shuffling tasks among available CPUs [44]. However, this dynamic task allocation strategy can introduce processing jitter, causing variability in the wait time for a process before it receives CPU time. Notably, the variability that processing jitter brings about may lead to missed deadlines or unforeseen delays in executing RT functions, potentially compromising the vBBU’s overall performance and, consequently, the quality of service in the mobile network.

CPU affinity provides a mechanism to designate which CPUs a particular process can utilize, providing a potential solution to minimize processing jitter [58,59]. By binding RT tasks—such as those within vBBU—to a specific group of CPUs, CPU affinity ensures that the Kernel Scheduler restricts CPU-time allocation to these assigned CPUs [60]. This strategy leverages the principle of processor cache locality, maintaining frequently used data in the cache of the specific CPUs, resulting in a more predictable and deterministic execution of RT tasks [61,62].

**Figure 4:**
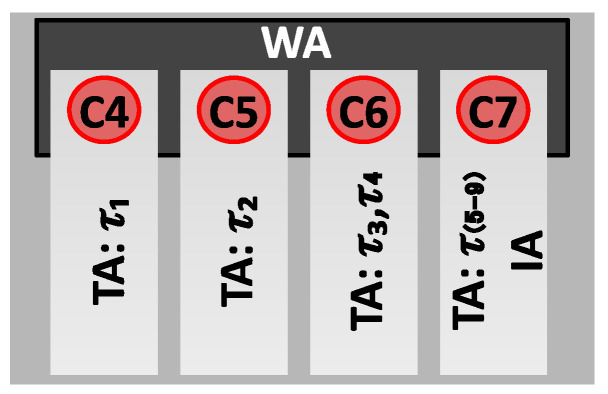
CPU affinity allocation strategies (*WA*, *TA*, and *IA*) for the vBBU.

For the purposes of our study, we explored three distinct implementations of CPU affinity, each representing a different level of CPU allocation strictness, which confines tasks to specific CPUs, as depicted in Figure 4. Let C={C0,…,C7} be the set of available CPUs in this specific network scenario. Furthermore, let $T=\{\tau_{1},\ldots ,\tau_{9}\}$ be the set of vBBU threads shown in Table 1. Assuming CPU affinity allocation to the vBBU on the CPUs {C4,C5,C6,C7} of *C*, the CPU affinity allocation scenarios for the vBBU threads in *T* are defined as follows:

In the first scenario, named Wide Affinity (*WA*), static CPU affinity is assigned to the vBBU on a predetermined subset of CPUs, denoted as $C_{W}\subset C$. Specifically, $C_{W}=\left\{C4,C5,C6,C7\right\}$.

In the second scenario, denoted Thread Affinity (TA), static CPU affinity is assigned per process thread (i.e., per child process) on a predetermined subset of CPUs, denoted as $C_{T}\subset C$. The CPU affinity allocation for each vBBU’s thread $\tau_{i}\in T$ is defined as $C_{\tau_{i}}\in C_{T}$ for each $\tau_{i}\in T$. Specifically, the TA scenario involves grouping related RT threads to run on the same CPU. Given that ru_thread ($\tau_{1}$) is the most CPU-intensive thread handling time domain IQ signal samples to the RRU, $\tau_{1}$ is assigned the CPU affinity $C_{\tau_{1}}=C4$ with no other thread on that CPU. Similarly, as lte-softmodem ($\tau_{2}$) is the second-most CPU-intensive thread dealing with L1 and L2 functions, $\tau_{2}$ is given the CPU affinity $C_{\tau_{2}}=C5$ without any other thread on that CPU. Notably, fep_processing ($\tau_{3}$) and feptx_thread ($\tau_{4}$) are related, both performing front-end processing for RX and TX, respectively. Hence, $\tau_{3}$ and $\tau_{4}$ share the CPU affinity $C_{\tau_{3}}=C_{\tau_{4}}=C6$. Allocating CPU affinity to all non-RT threads on a different CPU than the RT threads helps avoid unpredictable waiting times for the non-RT threads. Therefore, {$\tau_{5},\tau_{6},\tau_{7},\tau_{8},\tau_{9}\}$ are given by the CPU affinity $C_{\tau_{5}}=C_{\tau_{6}}=C_{\tau_{7}}=C_{\tau_{8}}=C_{\tau_{9}}=C7$.

These initial two CPU affinity scenarios aim to explore the benefits of assigning latency-sensitive vBBU threads to dedicated CPUs compared to the alternative approach, where the RT-Kernel dynamically schedules these threads across a predefined set of CPUs.

The third scenario, called Hard-IRQ CPU-affinity (IA), consists of hard-IRQs being assigned specific CPU affinities in order to optimize interrupt handling. Most modern NICs support multi-queue RX/TX. By default, an NIC’s driver instantiates as many RX/TX queues as CPUs in the system. By pinning each of these queues to a CPU, the driver registers a Hard-IRQ per queue. An incoming packet is copied to one of these RX-queues according to the traffic flow to which it belongs. Then, the driver raises the corresponding RX-queue’s Hard-IRQ or Soft-IRQ to process the packet following the Hard-IRQ or NAPI mechanism.

As demonstrated empirically in [54], assigning a specific CPU affinity to NIC’s RX/TX queue Hard-IRQs has been shown to effectively reduce packet processing latency in the Linux RT-Kernel. Building on this insight, we implement CPU affinity allocation to the RX/TX queue Hard-IRQs associated with the NIC port utilized by the vBBU as part of the Backhaul in the network scenario illustrated in Figure 1. Let $Q=\{q_{1},\ldots ,q_{7}\}$ represent the set of RX/TX queue Hard-IRQs defined by the NIC’s controller, with each of these RX/TX queue Hard-IRQs allocated the CPU affinity $C_{q_{i}}=C7,\;\forall q_{i}\in Q$.

The rationale behind allocating CPU affinity to the NIC’s RX/TX queue Hard-IRQs on CPU C7 is twofold. Firstly, this aligns with the CPU affinity allocation to the non-RT thread TASK_GTPV1_U ($\tau_{5}$) specified in the TA scenario. This strategic alignment not only reduces packet processing latency in the RT-Kernel by pinning the NIC’s RX/TX queue Hard-IRQs to a specific CPU but also optimizes the processing of packets consumed by the user-space process [63]. Essentially, $\tau_{5}$ represents the vBBU’s thread responsible for consuming Backhaul packets.

To evaluate the impact of CPU affinity allocation to the NIC on vBBU performance, the IA CPU allocation is integrated with the vBBU CPU affinity allocation in two scenarios: IA-WA, which combines the CPU affinity allocation to the NIC as in IA with the CPU affinity allocation to the vBBU according to WA; and IA–TA, which merges the CPU affinity allocation to the NIC as in IA with the CPU affinity allocation to the vBBU according to TA.

### 3.5. Mitigating Impact on vBBU RT Performance: Exploring CPU Isolation with Collocated Processes

CPU isolation is a well-known practice in RT systems aimed at preventing process interference [17]. This technique involves dedicating a specific subset of CPUs exclusively to a particular process, ensuring that no other user-space processes, and, in certain scenarios, no Kernel threads, are allocated CPU time on the same subset of CPUs. Implementing CPU isolation in this manner helps RT systems avoid processing interference from collocated workloads, contributing to improved performance and predictability.

We explore two CPU isolation approaches for the vBBU: Shielding (soft isolation) and isolCPU (hard isolation). Shielding involves isolating a specific subset of CPUs from user-space processes not intended to run within the shield. This isolation is achieved through the Kernel’s cpuset subsystem [64], which assigns individual CPUs and memory nodes to control groups (Cgroups). Resources allocated to Cgroups are only visible to the Cgroup members or their parents, meaning that certain system Kernel threads may not be moved outside the shield. Thus, Shielding is categorized as a soft CPU isolation approach. Our methodology for studying the vBBU under isolated CPUs using the Shielding approach involves adopting the CPU affinity allocation strategies IA-WA and IA-TA.

In contrast, isolCPU provides a more stringent form of CPU isolation by completely excluding a subset of CPUs from the RT-Kernel Scheduler [65]. Configured at boot time using the isolcpus feature [66], isolated CPUs cannot be allocated to any user-space process or Kernel-space threads, unless the CPU affinity of a process specifies that it should run on the isolated CPUs [17]. Because isolCPU fully prevents the Kernel Scheduler from allocating CPU time to isolated CPUs, it is considered a hard isolation approach [67]. Consequently, only specified processes can run on isolated CPUs, achieved by allocating CPU affinity. The methodology for studying the vBBU when running on isolated CPUs through the isolCPU approach consists of adopting CPU affinity allocations IA-WA and IA-TA.

In this section, we have outlined a comprehensive system model tailored to evaluate resource sharing and CPU management for executing vBBUs on edge servers. Our analysis incorporated the implementation of CPU affinity and isolation strategies aimed at mitigating the impact of resource contention on vBBU performance. This model serves as the cornerstone for our empirical evaluation, laying the groundwork for the subsequent performance assessments outlined in this paper.

## 4. Assessing the vBBU Performance in the Linux Kernel

This section conducts an empirical study on the performance of vBBU processes within the Linux RT-Kernel, instantiated as detailed in the system model in Section 3. The primary objective is to investigate the implications of resource sharing. Additionally, we delve into the application of CPU management strategies—specifically, CPU affinity and CPU isolation—with the aim of improving RT performance and mitigating processing interference.

### 4.1. Methodology

The methodology employed to assess the vBBU performance in the Linux RT-Kernel involves evaluating the vBBU performance for each of the resource sharing and CPU management strategy scenarios described in Section 3.

#### 4.1.1. Experimental Design

A series of experiments were conducted on the mobile network testbed scenario (Figure 3). Table A1 (please refer to Appendix A) summarizes the software and hardware specifications used to deploy this experimental setup. Utilizing the synthetic benchmark tool Iperf3 [68], a TCP flow with a target data rate of 10 Mbps was transmitted from an Iperf3 server situated outside the mobile network to an Iperf3 client at the UE.

For each of the resource-sharing and CPU-management scenarios outlined earlier, we carried out a series of six experiments, each of which lasted 10 min, resulting in a total observation period of 60 min. The duration of each experiment was selected to collect a significant number of data points that would be statistically significant for our analysis.

As outlined in the system model in Section 3, the User scenario is designed to simulate a situation where the vBBU shares computing resources with non-RT user-space processes. To achieve this, we used the synthetic benchmark tool stress ng [69] to generate workloads on various subsystems of the host machine. For example, we executed stress-ng instances on each CPU to perform I/O operations, stress the virtual memory, and write/read on disk (stress-ng -d 1 –hdd-bytes 200M -m 1 –vm-bytes 1G –iomix 1 –iomix-bytes 100M –mq 0.)

In the Kernel scenario, vBBU processes are executed alongside non-preemptive Kernel threads. As mentioned earlier, this scenario considers the processing of Hard-IRQs generated from incoming network packets as a use case for Kernel thread processing. To induce a high number of Hard-IRQs in the RT-Kernel, we introduce high packet-rate background traffic (BT) to the vBBU host edge server. The BT is generated by the Anritsu MT1000A Network Master Pro Tester [70] (referred to as the Anritsu device throughout the paper). Configured as a UDP flow with a frame size of 100 bytes and transmitted at 1000 Mbps (i.e., the maximum rate supported by the host’s NIC), BT uses a different NIC port than the one used by the vBBU in the Backhaul network. The host machine receives BT packets through an open UDP port. The Netcat network utility [71] serves as the receiver user-space application for BT packets. In this specific case, Netcat reads and writes packet content into the command line, generating additional workload on the edge server.

Additionally, to assess the effect of allocating CPU affinity to the NIC as defined for the IA scenario (see Section 3 for a system description on the CPU affinity), we conducted experiments to measure the RTT between the edge server hosting the vBBU and the Anritsu device. Specifically, we measured the RTT after sending ICMP packets (at a rate of one packet per second) to the NIC port used in the Backhaul, for which CPU affinity has been defined. The Anritsu device, which provides precise time synchronization for frame-delay measurements, was used to measure the RTT.

The methodology involves evaluating four scenarios. First, the noA scenario represents the default configuration where no CPU affinity has been defined for the NIC port used in the Backhaul. The second scenario, named IA, adopts CPU affinity allocation based on IA for the RX/TX queue Hard-IRQ of the NIC port used in the Backhaul. The remaining scenarios incorporate the BT flow. The inclusion of BT allows us to study latency when the Linux RT-Kernel utilizes the NAPI mechanism. The third scenario, noA-BT, combines the noA configuration on the NIC port used as Backhaul with the BT in a second NIC port. Finally, the fourth scenario, IA-BT, involves allocating CPU affinity based on IA to the Backhaul port, alongside the BT in the second port.

#### 4.1.2. Derived Metrics for Evaluation

To assess the performance of vBBU processes, we employ specific metrics that offer insights into their functionality. Two key metrics are considered for evaluation:

1. DL NACK: This metric is part of the DL Data Transmission Process (HARQ ACK/ NACK) [72]. If the UE detects an error in received DL data, it sends a DL NACK to the vBBU, triggering DL data retransmission. NACK counts, which indicate DL re-transmissions, provide valuable insights into the DL data path’s health;
2. Scheduling Latency: Measuring the waiting time a process undergoes to obtain CPU-time in the RT-Kernel, scheduling latency is a crucial metric that contributes to task processing latency [41]. Using the Kernel tracing tool BPF Compiler Collection [73], we assessed the scheduling latency for each vBBU thread outlined in Table 1.

### 4.2. Results and Discussion

Here, we present and analyze the results of our empirical study on vBBU performance within the Linux RT-Kernel, with a specific focus on resource-sharing and CPU-management strategies.

#### 4.2.1. Resource Sharing Impact

First, we analyze vBBU performance across the resource-sharing scenarios described in Section 3—Idle, User, and Kernel. The Idle scenario serves as our performance baseline, providing a reference point where the vBBU operates in isolation, without the influence of collocated user-space workloads or Kernel threads. By comparing the outcomes of the other two scenarios to the Idle scenario, we gain insights into how resource-sharing and CPU-management strategies impact the vBBU’s functionality and efficiency.

**Figure 5:**
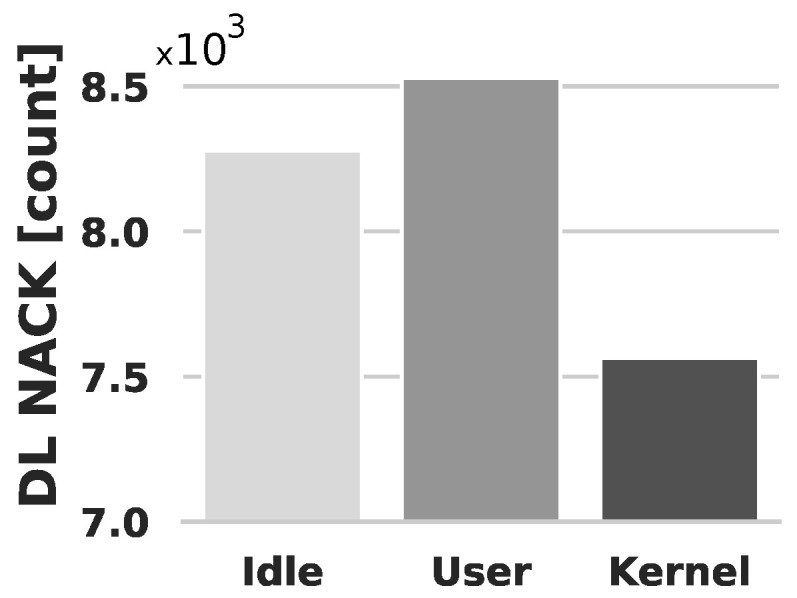
vBBU procedures: number of DL NACKs computed over a set of six experiments (total observation time-span is 60 min). Evaluated scenarios: (i) Idle (no resource sharing); (ii) User (sharing resources with collocated user-space workloads); (iii) Kernel (sharing resources with collocated Kernel threads).

The DL NACK metric is a crucial indicator of the health of the DL data path, where fewer NACKs imply a more robust path. Figure 5 presents the cumulative count of vBBU’s DL NACK for each scenario. Notably, the User scenario, where the vBBU operates alongside collocated user-space workloads, exhibits a 3% increase in DL NACK compared to the Idle scenario. This increase presumably stems from the additional strain on computing resources resulting from the concurrent execution of collocated user-space workloads alongside the vBBU.

In the Kernel scenario, the number of DL NACKs experiences a reduction of 8.6% compared to the Idle scenario. This decrease can be attributed to the efficient handling of high packet rates by the Linux RT-kernel. The Kernel scenario involves processing a high rate of incoming packets as a use case of Kernel thread processing. In the RT-Kernel, the handling of a high rate of incoming packets is achieved using NAPI, which polls and processes packets in batches within the context of Hard-IRQs.

The observation that the Idle scenario yields a higher count of DL NACKs compared to the Kernel scenario suggests that processing packets with a low incoming rate has a greater impact on the vBBU than processing packets with a high incoming rate in the RT-Kernel. In the Idle scenario, the edge server hosting the vBBU processes mobile traffic from the experiment, coming at a rate of 10 Mbps. Given that this traffic has a low enough packet rate to keep NAPI disabled, the NIC controller generates a Hard-IRQ for each arriving packet.

Table 2 provides information on the RT-Kernel scheduling latency events for the vBBU threads described in Table 1. The table categorizes events according to the percentage that falls into different latency buckets. In particular, it includes time intervals up to 15 microseconds, which covers more than 99% of all latency events.

In the Idle scenario, more than 93% latency events for all vBBU threads fall within the 0–1 microsecond interval. However, in the User scenario, where vBBU shares computing resources with collocated user-space workloads, there is a notable increase in scheduling latency for RT threads. Specifically, there is a shift in at least 35% of scheduling latency events to the 2–3 microsecond interval compared to the Idle scenario.

In the User scenario, where various subsystems like memory, disk, and I/O are stressed, there is a more pronounced impact on scheduling latency compared to the Kernel scenario. However, this impact differs among CPU-intensive threads, such as RT ru-thread and lte-softmodem, versus less intensive RT threads like fep_processing or feptx_thread. This difference can be attributed to their unique activity levels. Specifically, ru-thread and lte-softmodem consume more CPU time allocated to the vBBU compared to the fep_processing or feptx_thread, which have periods of inactivity ranging from 0.5–1 ms. Notably, the inactivity of the ru-thread never exceeds 64 μs. Thus, an increase in latency predominantly occurs during the wake-up of these inactive threads.

In the Kernel scenario, scheduling latency events for all vBBU threads exhibit higher latency compared to the Idle scenario. Even though this contrasts with the effect observed for NACKs, where the vBBU benefits from the efficient processing of high packet rates using NAPI compared to the Idle scenario, Kernel thread processing introduces a noticeable increase in the scheduling latency of the vBBU. This occurs because Hard-IRQ processing of incoming packets via Kernel threads cannot be preempted by the vBBU, causing prolonged wait times for it to access CPU-time.

Certainly, non-RT threads benefit from having more available CPUs in both idle and stressed systems. Since non-RT threads can be preempted by RT threads in the RT-Kernel, having more CPUs increases the opportunities for a non-RT thread to execute.

#### 4.2.2. CPU Management Strategies

This section assesses two distinct approaches aimed at mitigating the impact of sharing computing resources on the vBBU. Initially, we explore CPU affinity as a strategy to enhance the RT performance of vBBU latency-critical functions. Subsequently, we investigate the advantages of CPU isolation as a method to alleviate processing interference.

##### CPU Affinity

For each resource sharing scenario (i.e., Idle, User, Kernel), Table 3 displays scheduling latency events of vBBU threads under CPU affinity scenarios WA and TA. The table presents the percentage of events that fall into different latency buckets.

As mentioned earlier, the Idle scenario serves as a performance reference, as no other user-space processes are running on the edge server except the vBBU. Although there is no significant difference in the scheduling latency of RT threads when running the vBBU under WA or TA, non-RT threads experience higher scheduling latency in TA than in WA. The reason is that, in WA, the scheduler grants CPU time to the vBBU on any of the CPUs defined by the CPU affinity $C_{W}$. Conversely, when pinned to a fixed CPU, as in TA, a non-RT thread would experience an increased waiting time in the run queue until other tasks on the same CPU are descheduled.

In the User scenario, despite nearly half of vBBU’s RT threads experiencing a shift in scheduling latency events from the interval of 0–1 microseconds to 2–3 microseconds, the impact on the vBBU’s RT threads is less pronounced in TA than in WA. By pinning RT threads to a specific CPU in TA, these scheduling latency shifts are reduced by up to 8% compared to WA.

In the Kernel scenario, where the vBBU processes incoming packets in the context of Hard-IRQ, the impact on vBBU varies based on its thread characteristics, with critical threads such as ru_thread and lte-softmodem benefiting from TA, resulting in a 2% reduction in shifted latency events from the 0–1 microsecond interval to the 2–3 microsecond interval compared to WA. Conversely, RT threads fep_processing and feptx_thread experience an increase in scheduling latency under TA, with a 9% and 16% higher number of shifted latency events in the 2–3 microsecond interval than in WA, respectively. This increase is attributed to inactive periods experienced by these threads and the act of waking them up on the specific CPU they are pinned to in TA while Kernel threads are being scheduled.

In summary, when sharing computing resources with user-space workloads, vBBU’s RT threads benefit from TA CPU affinity as the impact on scheduling latency is lower than in WA. However, TA benefits only the two most demanding vBBU’s RT threads (ru_thread and lte-softmodem) in mitigating the impact of Kernel threads processing from incoming packet in the Linux Kernel. Unlike TA, where threads are pinned to a fixed CPU, WA circumscribes the vBBU’s threads to run on any of the CPUs in $C_{W}$, according to the Kernel’s scheduling policy. As a result, non-RT threads benefit from more available CPUs either on idle or loaded systems. Because non-RT threads might be preempted by RT threads in the RT-Kernel, the more CPUs, the more execution chances possessed by a non-RT thread.

Evidence suggests that allocating CPU affinity to the NIC’s RX/TX queue Hard-IRQs reduces packet processing latency in the Linux RT-Kernel [24]. Before analyzing the benefits of CPU affinity allocation based on IA to the vBBU performance, this section first studies the extent to which CPU affinity to the NIC mitigates packet processing latency by measuring the RTT between the edge server hosting the vBBU and the Anritsu device.

**Figure 6:**
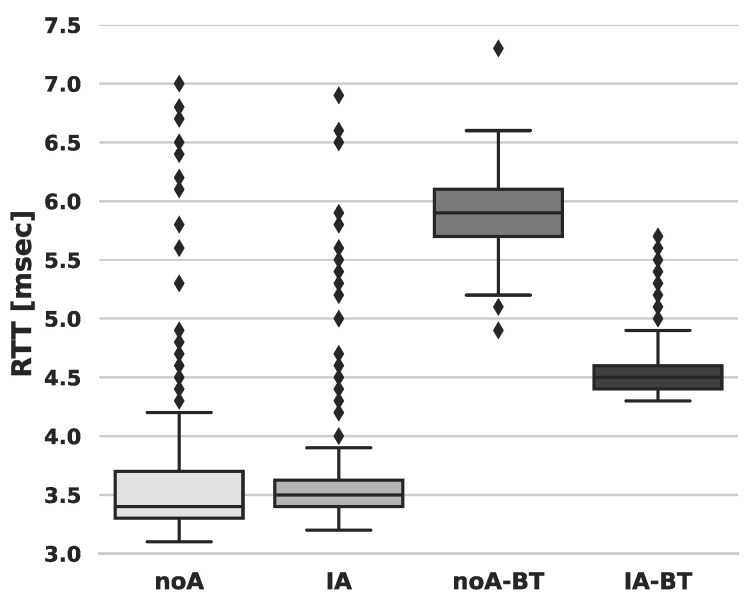
RTT estimate of packet processing latency in the RT-Kernel: comparing CPU affinity allocation to the NIC’s port RX/TX queue Hard-IRQ with no CPU affinity.

For each of the scenarios defined above, Figure 6 shows the RTT distribution. From these results, we make three observations about (i) the benefits of defining CPU affinity for the NIC; (ii) the increase in delay caused by the BT; (iii) the impact caused by per-packet processing through Hard-IRQ. For (i), the analysis is focused on the interquartile range. Defined as the difference (IQR=Q3−Q1) between the upper quartile (Q3: 75th percentile) and the lower quartile (Q1: 25th percentile) [74], the IQR provides insights into the central 50% of the data. The higher the IQR value, the more spread among the central 50% of the data. In this case, a wider distribution indicates a higher latency variation (jitter) of packet processing in the RT-Kernel. It is worth noting that low jitter and determinism are crucial for RT processes. For instance, the IQR for the two scenarios where no CPU affinity allocation is defined, noA and noA-BT, exhibits a wider spread, reaching up to 0.6.

In contrast, assigning CPU affinity to the Backhaul NIC port yields a more condensed distribution, consequently reducing jitter. The IQRs are 0.33 and 0.3 in IA and IA-BT, respectively. In essence, CPU affinity for the NIC reduces the spread of the data by half for 50% of the samples, compared to scenarios without CPU affinity.

In the presence of BT processing, there is an observable increase in packet processing delay (ii). Specifically, in the case of noA-BT, the RTT distribution experiences an increase of approximately 2.4 msec compared to noA, representing a substantial increase of 68%. Conversely, in IA-BT, the RTT distribution increases by around 1 msec, indicating a 28% rise compared to IA. This analysis effectively highlights the impact of BT processing on packet-processing delay and demonstrates the benefits of CPU affinity in mitigating this increase.

The boxplot outliers in the RTT distribution provide clear evidence of the impact caused by per-packet Hard-IRQ processing in the RT-Kernel (iii). Outliers, represented by data points outside the whiskers, highlight extreme values in the dataset. Whiskers, defined as the most extreme data points within the range of Q1 and Q3 and not greater than 1.5×IQR [8], help identify the presence of outliers.

In scenarios such as noA and IA, where the bit rate is low, preventing the activation of the NAPI mechanism, each packet is processed using Hard-IRQs, leading to unpredictable latency. The outliers observed in these scenarios are indicative of the unpredictable delays, potentially caused by factors like livelocks [75]. Notably, in noA, the outliers represent 9% of the data, while, in noA-BT, where NAPI is enabled due to the high packet rate flow of BT, outliers account for only 0.3% of the data. Similarly, in IA, outliers represent 11% of the data, while, in IA-BT, with NAPI enabled, outliers represent 6% of the data. This observation underscores the effectiveness of NAPI in mitigating unpredictable latency induced by per-packet Hard-IRQ processing.

Certainly, these results suggest that allocating CPU affinity to the NIC enhances the determinism of packet processing in the RT-Kernel. Now, the question is whether the vBBU would benefit from allocating CPU affinity to the NIC port used in the Backhaul. To answer this question, we next evaluate the scheduling latency of vBBU threads while incorporating IA into the CPU affinity allocation scenarios defined for the vBBU: IA-WA and IA-TA.

Table 4 presents the scheduling latency events of vBBU threads under CPU affinity allocations IA-WA and IA-TA. As this analysis centers on adopting CPU affinity allocation to the NIC port used in the Backhaul, we specifically evaluate the Kernel scenario, involving the sharing of computing resources with Kernel thread processing generated by incoming packets.

These results indicate that the vBBU benefits from allocating CPU affinity to the Backhaul port in mitigating the impact of packet processing in the Linux RT-Kernel. In both scenarios, IA-WA and IA-TA, scheduling latency events of vBBU RT threads shows an increase in the 0-1 interval compared to the results reported in Table 3, where no CPU affinity was allocated to the NIC. In other words, adopting IA to the Backhaul port helps alleviate the impact on the vBBU RT threads scheduling latency caused by packet processing.

As mentioned earlier, vBBU RT threads, namely, ru-thread and lte-softmodem, account for a significant portion of the CPU time allocated for the vBBU. In the configuration with both fixed CPU affinity per thread and CPU affinity to the NIC, as, in IA-TA, RT threads ru-thread and lte-softmodem experience lower scheduling latency compared to IA-WA. However, the impact on non-RT threads is the opposite. In IA-WA, non-RT threads can receive CPU time on any of the CPUs in $C_{W}$, resulting in lower scheduling latency for non-RT threads compared to IA-TA. Consequently, in IA-TA, latency events of non-RT threads even shift beyond the 2–3 microsecond range.

##### CPU Isolation

**Figure 7:**
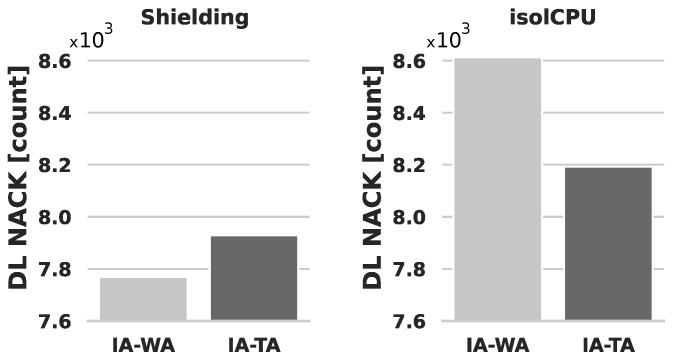
vBBU’s number of DL NACKs computed over a set of six experiments (total observation time-span is 60 min). Evaluated scenarios: Kernel–CPU affinity allocation: IA-WA vs. IA-TA.

Based on the evidence presented thus far, the coexistence of user-space processes and Kernel threads sharing computing resources has a noticeable impact on vBBU performance. In this section, we explore the effectiveness of two CPU isolation mechanisms, namely, Shielding and isolCPU, as potential solutions to mitigate the influence of concurrently executing workloads. Since CPU isolation ensures that all user-space processes run on CPUs other than isolated ones, scenarios such as Idle and User exhibit no direct impact on vBBU performance. Consequently, our evaluation focuses exclusively on the Kernel scenario. To gain deeper insights into the advantages of Shielding compared to isolCPU for the vBBU, we analyzed the performance of vBBU’s DL NACK procedures, as illustrated in Figure 7.

The Shielding approach demonstrates better performance by producing fewer DL NACKs compared to isolCPU. Remarkably, Shielding outperforms even the results observed in Figure 5 for the Idle scenario, where the vBBU utilizes all available CPUs in the edge server. In this case, the allocation of CPU affinity through IA-WA enhances vBBU performance in terms of DL NACKs, as opposed to IA-TA. In contrast, when employing CPU isolation with isolCPU, the vBBU experiences a higher number of DL NACKs for both IA-WA and IA-TA, surpassing even the performance observed in a stressed system, such as in the User scenario depicted in Figure 5. This discrepancy is attributed to the implications of isolating CPUs in isolCPU, which involves migrating buffered data chunks. Potentially, these chunks may be in the cache of CPUs outside the isolated set, leading to degradation in the DL data path.

Certainly, isolating CPUs mitigates the impact on vBBU scheduling latency caused by sharing computing resources. As shown in Table 5, both CPU isolation approaches result in lower vBBU scheduling latency compared to the results presented in Table 2, Table 3 and Table 4 for the Kernel scenario.

However, the specific benefit of isolCPU over Shielding in terms of vBBU scheduling latency is not entirely clear. The observed difference in scheduling latency results between the two cases is not significant. Some related research [63] suggests that traffic-based applications benefit from hard isolation, reducing processing latency. However, because the vBBU involves various operations beyond packet processing, such as I/O, such a benefit may not be readily apparent.

While isolating CPUs proves beneficial in mitigating the impact of sharing computing resources, it comes with the trade-off of resource underutilization. Therefore, before adopting this approach, a careful assessment of the trade-off between mitigating processing interference and maintaining resource efficiency is essential.

## 5. Assessing Resource Sharing in the Cloud-RAN Architecture

The Cloud-RAN architecture involves multiple vBBUs instantiated on the same edge computer, collectively forming what is known as the vBBU pool. Utilizing virtualization technology, particularly, containers in this study, these vBBUs operate as isolated processes. However, the presence of collocated containers or overloaded Kernel threads, such as those engaged in high-rate packet processing in the Linux Kernel, can have a notable impact on the performance of vBBUs. One contributing factor is the scheduler that allocates CPU time to processes from different containers on the same CPU.

To address this issue, we propose an orthogonal CPU affinity allocation to containers, utilizing the CPU affinity approaches *IA* and *WA*, as introduced in Section 3.

### 5.1. Mobile Network Scenario

**Figure 8:**
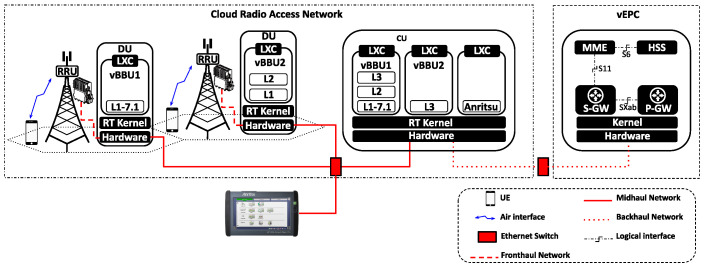
Mobile network scenario based on the Cloud-RAN architecture. Deploying two vBBUs with split 7.1 and split 2, respectively. The mobile transport Xhaul is based on switched Ethernet.

We consider the mobile network scenario illustrated in Figure 8. This configuration features two vBBUs, denoted as vBBU1 and vBBU2, respectively. Each vBBU, deployed with distinct functional splits, operates between the DU and the CU. While vBBU1 and vBBU2 are the only applications instantiated in the DU, they share computing resources at the CU. Moreover, this network scenario involves a single UE connected to each of the vBBUs. The experimental setup for the deployment of this mobile network is summarized in Appendix A.

Based on the LTE–BBU functional split model shown in Figure 2, vBBU1 adopts the functional split 7.1, while vBBU2 adopts the functional split 2. Due to split 7.1 hosting most of the BBU RT functions in the CU, vBBU1 imposes greater demands in terms of latency requirements in the Midhaul and processing capacity at the CU. In contrast, vBBU2 deploys all functions involved in the HARQ loop in the DU, making its data-rate requirements in the Midhaul network similar to those in the Backhaul. Here, latency requirements depend on the user’s service.

Linux containers (LXC) serve as the virtualization environment for deploying both vBBUs at the DU and CU. To assess resource sharing in Cloud-RAN, we introduce an additional service which involves a traffic flow generated by the Anritsu device and aggregated in the Midhaul. The destination for this traffic is a third LXC is deployed at the CU, referred to as A. Designed as a non-RT user-space application sharing computing and network resources with vBBUs at the CU, A consists of the network utility Netcat, which consumes packets from the traffic flow.

In a containerized virtualization, the NIC is likely shared among different containers. In this network scenario, all services instatiated at the CU—vBBU1, vBBU2, and A—share the NIC port connecting the CU to the Midhaul network. Similarly, vBBU1 and vBBU2 share the second NIC port connecting the CU to the Backhaul. Using two different NIC ports to access the Midhaul and the Backhaul allows for isolating traffic in the Midhaul with time requirements from traffic in the Backhaul, which does not impose any time requirements.

To facilitate NIC sharing among containers, the edge server associated with the CU deploys an SR-IOV-based NIC. This implementation leverages SR-IOV to create multiple virtual functions (VFs) atop the NIC’s PCI Express (PCIe) physical function (PF). The NIC’s driver supporting SR-IOV registers the corresponding RX/TX Hard-IRQ for that VF. Moreover, a unique MAC address is assigned to each VF. This pair of MAC addresses—RX/TX Hard-IRQ—makes the VF look like an independent NIC itself. Each VF is assigned a unique MAC address and is linked to corresponding RX/TX Hard-IRQs, which together make the VF appear as an independent NIC. Assigned to one of such VF, an LXC can access the network with complete traffic isolation and without relying on any Kernel features for the creation of vNICs assigned to containers or internal virtual switching for their operation. Previous evidence suggests that mechanisms based on creating virtual NIC (vNIC) like macvlan or SR-IOV provide lower overhead than Kernel-based software switch mechanisms like Linux bridge or Open vSwitch (OVS) [76].

**Figure 9:**
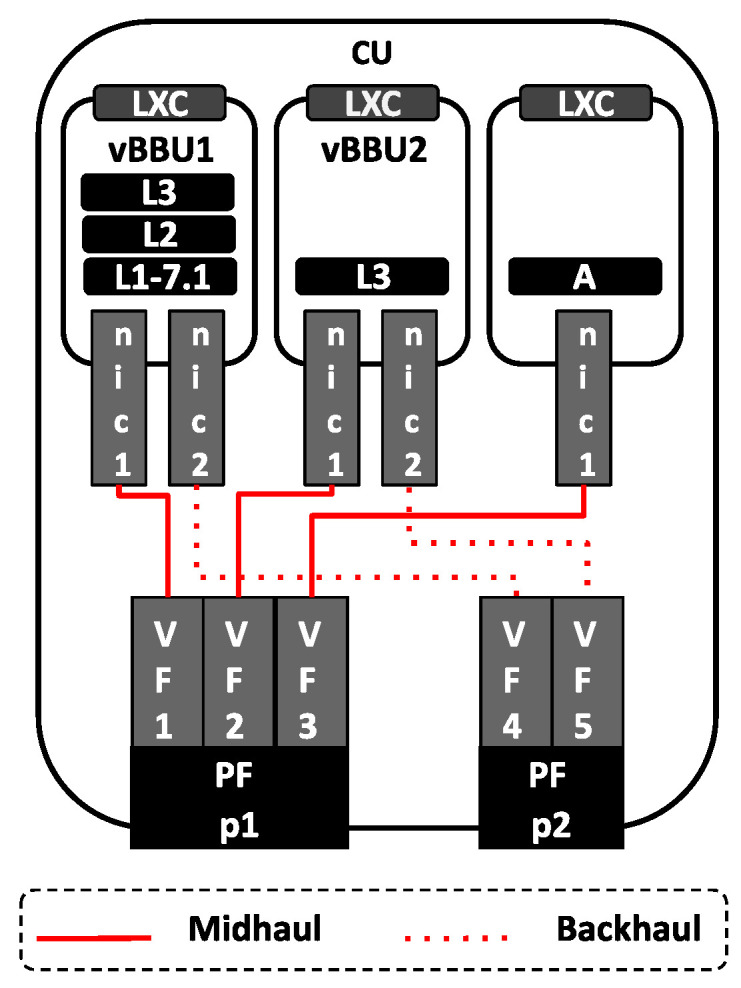
SR-IOV-based NIC sharing in Cloud-RAN-Deploying two different ports: p1 provides access to the Midhaul; p2 provides access to the Backhaul.

As depicted in Figure 9, the NIC used in this network scenario features two Ethernet ports that support SR-IOV (see the hardware details in Table A1). The first port, known as p1, is used in Midhaul. As all three LXCs deployed at the CU are connected to the Midhaul, three VFs are created on top of p1’s PF: VF1, VF2, and VF3. Each LXC defines a vNIC called nic1, with a different VF assigned to each LXC, allowing access to the Midhaul. For example, VF1 is assigned to the nic1 of vBBU1, VF2 to the nic1 of vBBU2, and VF3 to the nic1 of A.

On the other hand, the second NIC port, p2, is used in Backhaul. Since only vBBU1 and vBBU2 connect to the Backhaul, two VFs are created on top of p2’s PF: VF4 and VF5. Additionally, both vBBU1 and vBBU2 define a second vNIC called nic2. VF4 is assigned to the nic2 of vBBU1, while VF5 is assigned to the nic2 of vBBU2 for access to the Backhaul.

### 5.2. Sharing Computing Resources at the CU

In the network scenario evaluated in this section, three user-space applications share resources in the CU. Firstly, vBBU1, which deploys split 7.1, is the most time-critical application. Since vBBU1 shares frequency domain samples of the signal between the DU and the CU, it is sensitive to processing interference or resource unavailability. Evidence suggests that running vBBU1 on a minimum of four CPUs guarantees stable performance [50]. On the contrary, vBBU2, which implements split 2, hosts L3 functions at the CU. L3 functions are not time-critical and, therefore, run as non-RT threads in the Linux RT-Kernel. Consequently, in this deployment, two CPUs are assigned to vBBU2. Lastly, service A runs as a best-effort application for BT packets. Thus, no more than a single CPU is assigned to LXC hosting A.

**Figure 10:**
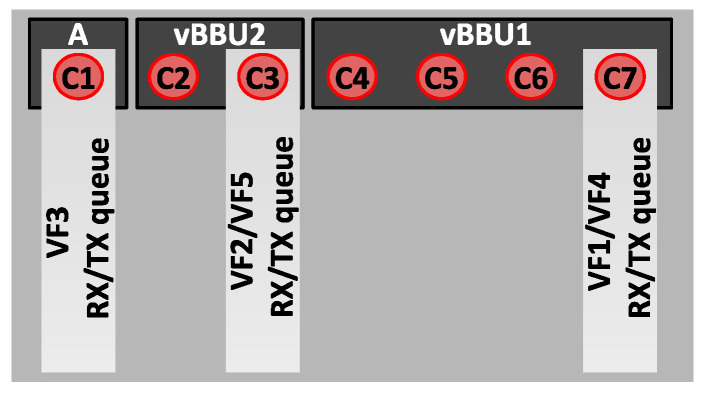
CPU affinity allocation based on *IA-WA* to the vBBU and to the vNIC VF’s RX/TX queue Hard-IRQ.

To mitigate processing interference from collocated LXCs, we assess the CPU affinity scenario *IA-WA* introduced in Section 3. Figure 10 shows the CPU affinity assigned to the LXCs hosted at the CU. Following the *WA* approach, while the CPU affinity to vBBU1 is $C_{W}^{BBU1}=\left\{C4,C5,C6,C7\right\}$, the CPU affinity to vBBU2 is defined as $C_{W}^{BBU2}=\left\{C2,C3\right\}$. Similarly, the CPU affinity to A is $C_{W}^{A}=\left\{C1\right\}$. In this way, by pinning LXCs to disjoint subsets of CPUs, user-space process isolation is guaranteed.

On the other hand, following the *IA* approach, we adopted the CPU affinity to the NIC. This time, the CPU affinities are allocated to the SR-IOV’s VFs, as shown in Figure 10. More specifically, the RX/TX Hard-IRQ queues associated with VF1 and VF4 in vBBU1 are allocated CPU affinity $C_{VF1}^{BBU1}=C_{VF4}^{BBU1}=C7$. Similarly, the RX/TX Hard-IRQ queues associated with VF2 and VF5 in vBBU2 are allocated CPU affinity $C_{VF2}^{BBU2}=C_{VF5}^{BBU2}=C3$. Finally, the RX/TX Hard-IRQ queue associated with VF3 in A is allocated CPU affinity $C_{VF3}^{A}=C1$. This way, high-rate incoming packets and their corresponding Hard-IRQs or Soft-IRQs (in NAPI) are processed by a different CPU than the ones used by the vBBUs, thus avoiding processing interference.

### 5.3. Results and Discussion

Two scenarios of resource sharing have been considered for evaluation: (i) noA, where no CPU affinity is defined. This scenario provides a baseline for the evaluation, where the RT-Kernel Scheduler decides to which CPUs mapping the resources assigned to a LXC. Such a decision could vary over time depending on the system load, resource availability, and process priorities; (ii) IA-WA, which adopts the CPU affinity approach described above for all LXCs and vNICs. Here, the CPU mapping is predefined according to CPU affinities.

Conducted experiments consist of measuring end-to-end throughput at each UE connected to a given vBBU. In this case, the end-to-end throughput is derived from a TCP downstream flow generated by an Iperf3 server, which is located right outside the mobile network. The destination of the TCP flow is an Iperf3 client instantiated at the UE. A set of six experiments of 10 min each is conducted per UE. In total, two downstream TCP flows, one per UE, are generated with an initial rate of 10 Mbps. While conducting an experiment for a given UE, the TCP downstream flow for the second UE runs as background traffic. In addition, the BT from the Anritsu device to the A service at the CU runs during the whole experimentation. Not only does the BT allow for evaluating traffic aggregation and network sharing in the Midhaul, but the BT’s destination service A also allows evaluating resource sharing at the CU.

**Figure 11:**
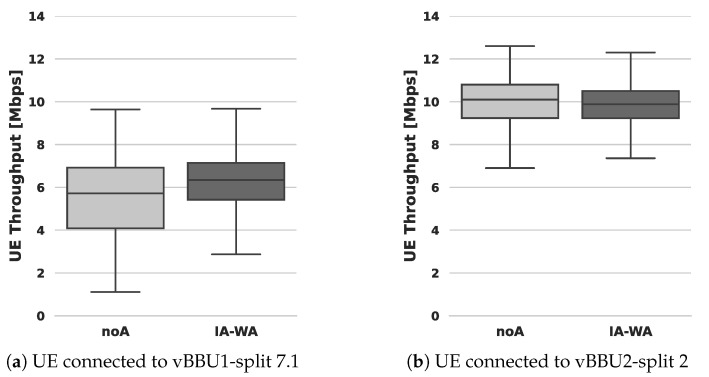
Cloud-RAN performance: end-to-end throughput measured by vBBU’s UEs. CPU affinity allocation: (i) noA vs. (ii) IA-WA.

Figure 11 shows the results after computing the end-to-end throughput as measured by the UE’s Iperf3 client.

The advantages of implementing the proposed CPU affinity are evident in the distribution spread of the data illustrated in Figure 11. Particularly notable is the substantial benefit for the UE connected to vBBU1. In the IA-WA scenario, the median UE’s received throughput reaches 6.7 Mbps, outperforming the noA scenario where the median throughput is 5.7 Mbps. Moreover, the Interquartile Range (IQR), representing the range in which 50% of the sample data fall, is reduced from 4.2 in noA to 2.5 in IA-WA. This indicates a 40% reduction in throughput variability achieved by allocating CPU affinity based on IA-WA in comparison with scenarios without CPU affinity.

Although the impact of adopting CPU affinity is not as pronounced for vBBU2 with split 2 as it is for vBBU1, there remains a noticeable difference in the distribution spread compared to the noA scenario. As depicted in Figure 11b, for the UE connected to vBBU2, the IQR is 1.9 in the IA-WA scenario, whereas it is 2.3 in the noA scenario. In other words, by allocating CPU affinity based on IA-WA, the throughput variability is reduced by 21% in comparison with scenarios without CPU affinity.

## 6. Conclusions

This study provides insights into the impact of resource sharing with collocated workloads on vBBU performance in edge servers managed by the Linux Kernel. The empirical investigation highlights the substantial challenges posed by resource contention when vBBUs share resources with user-space tasks and Kernel threads.

Effective CPU management strategies, particularly, CPU affinity, emerge as crucial mechanisms for significantly enhancing vBBU performance. The application of CPU affinity, demonstrates substantial benefits, including a notable reduction in throughput variability, decreased vBBU NACK ratios, and lowered scheduling latency within the Linux RT-Kernel.

Although CPU isolation exhibits performance comparable to that in a reference scenario where vBBU is the only active process, the trade-off involves the potential underutilization of resources. In contrast, the allocation of orthogonal CPU affinity to containers hosting both vBBUs and best-effort applications demonstrates the potential to guarantee CPU isolation, thereby improving end-to-end vBBU performance in a Cloud-RAN setup.

Looking ahead, the static nature of CPU affinity allocation in this study prompts a call for future exploration into dynamic assignment methodologies. Future research efforts will focus on adapting CPU affinity allocations based on real-time application demands, CPU usage, and availability, with a view to improving adaptability and responsiveness to varying workloads. Additionally, the research agenda includes the investigation of resource contention scenarios in diverse setups, such as those involving dynamic functional splitting within Cloud-RAN. The ultimate goal is to develop robust strategies and mechanisms that optimize vBBU performance across a spectrum of Cloud-RAN configurations.

While our investigation anchors itself primarily on the exploration of CPU resource sharing and its direct impact on vBBU performance, we recognize the vast landscape of potential performance-affecting factors within a Cloud-RAN environment. Future studies could further this research by considering a more diverse array of performance metrics and external factors that contribute to system efficiency.

As part of our future work, we look forward to comparisons with alternative resource management strategies to ascertain the distinct advantages of CPU affinity and CPU isolation within Cloud-RAN deployments. This study lays the groundwork by establishing a benchmark for the effectiveness of these well-understood strategies in managing vBBU performance in resource-sharing scenarios. We recognize the limitations arising from the scope of experimentation at our disposal and hope that future endeavors could expand upon our work, contributing further to the complex discussion of resource management in real-time systems.

Although the results we have presented provide valuable insights into the performance dynamics of vBBUs within our experimental configuration, we fully recognize that these findings are contingent on the specific conditions and constraints of our setup. These results serve as a snapshot of performance under certain hardware specifications, network conditions, and software settings, providing a focused examination that is inherently limited in scope. Consequently, the broader applicability of our findings to different configurations warrants caution and further investigation. Moving forward, future research could benefit from a diverse range of experimental scenarios, including varying hardware platforms and network loads, to enhance the generalizability of results and provide a more versatile understanding of CPU management strategies in varied Cloud-RAN environments.

While our study successfully establishes the impact of resource sharing on vBBU performance and illustrates the utility of CPU affinity and isolation as mitigation strategies, we acknowledge that transitioning these strategies to a large-scale Cloud-RAN deployment presents its own set of challenges. The scalability of these CPU management techniques and their adaptability within a dynamic and heterogeneous network environment remain crucial factors for real-world implementation. Future work could specifically focus on the practical aspects of deploying these strategies, with an emphasis on automation, scalability, and management within broader Cloud-RAN frameworks.

## Figures and Tables

**Table 1:** OAI’s LTE–vBBU functions instantiated as threads (subprocesses) in the Linux RT-Kernel.

Thread	Time Requirement	Description	Index
ru-thread	RT	Radio unit processing	$\tau_{1}$
lte-softmodem	RT	L1–L2 processing	$\tau_{2}$
fep_processing	RT	Front End Process—RX	$\tau_{3}$
feptx_thread	RT	Front End Process—TX	$\tau_{4}$
TASK_GTPV1_U	non-RT	GTP tunneling	$\tau_{5}$
TASK_UDP	non-RT	UDP socket	$\tau_{6}$
TASK_S1AP	non-RT	S1 channel	$\tau_{7}$
TASK_SCTP	non-RT	SCTP channel	$\tau_{8}$
TASK_RRC	non-RT	RRC channel	$\tau_{9}$

**Table 2:** Scheduling latency of vBBU’s threads (percentage). Evaluated scenarios: (i) Idle (no resource sharing); (ii) User (sharing resources with collocated user-space workloads); (iii) Kernel (sharing resources with collocated Kernel threads).

Thread	0–1 (microseconds)			2–3 (microseconds)			4–7 (microseconds)			8–15 (microseconds)		
	Idle	User	Kernel	Idle	User	Kernel	Idle	User	Kernel	Idle	User	Kernel
ru-thread	93.8	53.1	90.9	0.4	41.9	4.8	0.1	1	0.3	5.7	4	3.9
lte-softmodem	99.4	49.5	91.6	0.3	49.1	7.8	0.01	0.7	0.2	0.3	0.6	0.4
fep_processing	99.3	63	89.7	0.4	35	9.3	0.05	1.1	0.5	0.3	0.9	0.4
feptx_thread	99.4	51.4	76.1	0.5	44.8	23	0.03	3.5	0.7	0.03	0.1	0.1
TASK_GTP	98.6	58.6	84.8	0.8	38.1	10.7	0.4	2	3	0.2	1.1	1.2
UDP_TASK	95.2	52	77.9	1	41.4	12.9	2.8	4.4	3.7	0.8	2	1.8

**Table 3:** Scheduling latency of vBBU’s threads (percentage). Evaluated scenarios: (i) Idle (no resource sharing); (ii) User (sharing computing resources with collocated user-space workloads); (iii) Kernel (sharing computing resources with collocated Kernel thread processing-packet processing use case)-CPU affinity allocation: WA vs TA.

Thread	0–1 (microseconds)						2–3 (microseconds)						4–7 (microseconds)						8–15 (microseconds)					
	Idle		User		Kernel		Idle		User		Kernel		Idle		User		Kernel		Idle		User		Kernel	
	WA	TA	WA	TA	WA	TA	WA	TA	WA	TA	WA	TA	WA	TA	WA	TA	WA	TA	WA	TA	WA	TA	WA	TA
ru-thread	93.5	93.3	45.7	52.9	78.8	81.6	0.4	0.3	49.2	41.1	17.2	15.4	0.1	0.1	1.4	1.7	0.7	0.6	5.9	6.2	3.6	4	3.3	2.3
lte-softmodem	99.3	99.1	28.6	49.2	74.2	80.9	0.5	0.3	57.1	49	19.3	17.4	0.03	0	14.3	0.6	0	0.9	0.2	0.1	0	0.8	6.4	0.4
fep_processing	98.8	99.2	45.4	56.7	70.3	60.1	0.5	0.2	46.8	39.7	25	33.8	0.02	0	5.5	1.8	3.1	4.3	0.5	0.6	2	1.7	1.42	1.6
feptx_thread	99.3	99.7	47	53.4	72	54.4	0.7	0.3	49.7	45.6	26.2	42.8	0.05	0	3	0.9	1.7	2.2	0.02	0	0.2	0.1	0.1	0.4
TASK_GTP	94.4	6.4	46.9	2.3	75.6	1.04	0.8	86.3	43.8	89.6	17.9	90.5	3.3	6	4.9	3.3	3.9	3.9	1.3	1	3.8	4.4	2.3	4.3
UDP_TASK	95.4	90.8	22.9	38.7	57.4	51.3	1.4	6.2	65.2	53.5	35.8	40.7	2	1.3	7.8	5.4	4.4	3.4	1	1	3.7	1.5	2.1	3.8

**Table 4:** Scheduling latency of vBBU’s threads (percentage). Evaluated scenarios: (i) Kernel (sharing computing resources with collocated Kernel thread processing generated by incoming packets)—CPU affinity allocation: IA-WA vs. IA-TA.

Thread	0–1 (microseconds)		2–3 (microseconds)		4–7 (microseconds)		8–15 (microseconds)	
	Kernel		Kernel		Kernel		Kernel	
	IA-WA	IA-TA	IA-WA	IA-TA	IA-WA	IA-TA	IA-WA	IA-TA
ru-thread	89.4	90.8	6.5	4.5	0.4	0.34	3.5	4.3
lte-softmodem	81	93.4	7.2	5.4	0.8	0.2	1.5	0.3
fep_processing	86.3	80.2	11.2	17.7	0.6	0.8	0.8	1.2
feptx_thread	72.9	75.9	15.6	23.4	1.2	0.4	1.4	0.1
TASK_GTP	74.6	0.1	9.3	56.4	4.7	32.6	2.2	4.5
UDP_TASK	62.5	49.4	6.2	2.7	23	32.5	3.5	14.2

**Table 5:** Scheduling latency of vBBU’s threads (percentage). Evaluated scenario: Kernel (sharing computing resources with collocated Kernel thread processing generated by incoming packet processing). CPU affinity allocation: IA-WA vs. IA-TA; CPU isolation: Shielding vs isolCPU.

Thread	0–1 (microseconds)				2–3 (microseconds)				4–7 (microseconds)				8–15 (microseconds)			
	Shielding		isolCPU		Shielding		isolCPU		Shielding		isolCPU		Shielding		isolCPU	
	IA-WA	IA-TA	IA-WA	IA-TA	IA-WA	IA-TA	IA-WA	IA-TA	IA-WA	IA-TA	IA-WA	IA-TA	IA-WA	IA-TA	IA-WA	IA-TA
ru-thread	92.8	93.9	92	93.1	0.3	0.3	0.3	0.3	0.2	0.2	0.2	0.3	6.4	5.4	7.1	6.2
lte-softmodem	98.6	98.4	100	98.4	0.5	0.4	0	0.4	0.2	0.2	0	0.2	0.4	0.4	0	0.4
fep_processing	98.1	98.3	98.1	98.5	0.7	0.4	0.7	0.2	0.1	0.1	0.2	0.1	0.8	1	0.9	1
feptx_thread	98.7	99	98.5	99	0.6	0.4	0.9	0.4	0.2	0.1	0.3	0.1	0.2	0.2	0.2	0.2
TASK_GTP	96.8	93.5	96.8	93.5	0.7	4.7	0.7	4.7	1.6	1.2	1.6	1.2	0.6	0.2	0.6	0.2
UDP_TASK	92.6	88.4	92.4	89	1	5.9	1	6	4.8	4.4	4.8	3.7	1.4	0.7	1.3	0.7

## Data Availability

Data are contained within the article.

## Appendix A. Testbed Setup Specifications

Table A1 provides an overview of the hardware and software specifications for deploying the experimental setup corresponding to the mobile network scenarios depicted in Figure 3 and Figure 8.

**Table A1:** Testbed setup for the mobile network scenario corresponding to both the monolithic vBBU deployment in Figure 3 and the Cloud-RAN deployment in Figure 8—hardware and software specifications.

Component	Description
UE	OnePlus-5 phone/Software: OxygenOS Version 10.0.1/Oneplus, Shenzhen, China.
air interface (as specified by the vBBU)	25 Physical Resource Blocks (PRB), which provides 5 MHz bandwidth.
RRU	Ettus (B210) One antenna port—Single Input Single Output (SISO)/Software: Universal Software Radio Peripheral (USRP) Version: 4.6.0.0-7-gece7c4811/Ettus Research, Austin, TX, USA.
Fronthaul	Fast SuperSpeed USB 3.0 connectivity at 5.0 Gbit/s (provided by Ettus (B210)).
Monolithic vBBU deployment (see Figure 3)	
vBBU	Monolithic eNodeB-LTE implementation from OpenAirInterface (OAI) Version 2.0.0.
Edge server	Equipped with eight Intel i7-8750H physical processors at 2.20 GHz, and 32 GiB of memory.
Edge server’s OS	Ubuntu 18.04 with low-latency Linux kernel version 5.3.28.
Edge server’s NIC	Supermicro AOC-SG-i2 Gigabit Ethernet adapter, equipped with two Intel 82575 Gigabit Ethernet ports/San Jose, CA, USA.
Backhaul	physical link at 1 Gbit/s.
Cloud-RAN (see Figure 8)	
vBBU1	OpenAirInterface eNodeB–LTE implementation Version 2.0.0, with split 7.1. This functional split uses the NGFI-IF4p5 interface specification [77]
vBBU2	OpenAirInterface eNodeB–LTE implementation Version 2.0.0, with split 2 using the F1 Application Protocol (F1AP) [78,79].
DU	Intel NUC7i7BNB equipped with four Intel Core i7-7567U processors, and 32 GiB of memory.
DU’s OS	Ubuntu 16.04 with low-latency Linux kernel version 4.19.58.
CU	Edge server equipped with eight Intel i7-8750H physical processors at 2.20 GHz, and 32 GiB of memory.
CU’s OS	Ubuntu 20.04 with Linux Kernel low-latency patch version 5.4.0.125.126.
Midhaul & Backhaul	Juniper EX4200 Ethernet switch, with physical interfaces at 1 Gbit/ Software: Junos OS Version 12.3R12-S21.
Core Network	
vEPC	4G EPC implementation from OAI.
vEPC’s host physical machine	Generic server equipped with four Intel i7 processor at 2.20 GHz, and 12 GiB of memory.
vEPC’s host OS	Ubuntu 18.04 with generic Linux kernel version 5.3.28.
